# Early Neutrophil Activation in Psoriatic Skin at Relapse Following Dead Sea Climatotherapy

**DOI:** 10.1111/exd.70094

**Published:** 2025-04-03

**Authors:** Thomas Emmanuel, Hakim Ben Abdallah, Elena Baez, Ida Maja Rather, Torben Steiniche, Anne Bregnhøj, Lars Iversen, Claus Johansen

**Affiliations:** ^1^ Department of Dermatology Aarhus University Hospital Aarhus Denmark; ^2^ Department of Clinical Medicine Aarhus University Hospital Aarhus Denmark; ^3^ Department of Pathology Aarhus University Hospital Aarhus Denmark; ^4^ MC2 Therapeutics A/S Hoersholm Denmark

**Keywords:** Dead Sea climatotherapy, disease memory, psoriasis, relapse, transcriptomics

## Abstract

Psoriasis, a chronic inflammatory skin disorder characterised by erythematous and scaly plaques, can be both physically and emotionally distressing for patients. Dead Sea climatotherapy (DSC), a treatment modality combining sun exposure, mineral‐rich water and mud therapy during 4 weeks at Ein Gedi, Israel, is used for a small group of patients with psoriasis. This study aimed to investigate the cellular composition of psoriatic skin lesions at relapse after complete clearance from DSC. Skin biopsies from baseline, end of treatment and relapse were collected from eight patients with plaque psoriasis who achieved complete clearance from Dead Sea climatotherapy treatment. These biopsies were subjected to immunohistochemistry, RNA sequencing and quantitative polymerase chain reaction analysis (qPCR). Our findings demonstrate that DSC effectively reduces inflammatory markers to levels comparable to baseline non‐lesional skin in the short term. The differential expression analysis identified several upregulated differentially expressed genes, including *OSM*, *CXCL8*, *TREM1*, *CXCL1*, *CSF3R*, *BCL2A1* and *CXCL2*, in relapsed psoriasis skin compared with baseline lesional skin. These findings were confirmed by qPCR analysis. Pathway enrichment analysis indicated a marked upregulation of neutrophil‐associated pathways in relapse skin compared with baseline lesional skin. Immunohistochemical staining for neutrophil markers, such as CD11b, CD15, CD66b, CD207, MPO and NE, showed a non‐significant trend towards enhanced neutrophil infiltration and activation at relapse. In conclusion, while DSC provides short‐term effectiveness in managing psoriasis, the initial relapse phase is associated with neutrophil activation and migration. Thus, targeting neutrophils early in the psoriasis disease course may disturb the evolution of psoriasis, potentially preventing disease chronicity.

**Abbreviations**
CD: cluster of differentiation
DEG: differentially expressed gene
DSC: Dead Sea climatotherapy
EOT: end of treatment
GPP: generalised pustular psoriasis
GSVA: gene set variation analysis
IL: interleukin
LS: lesional
MPO: myeloperoxidase
NE: neutrophil elastase
NL: non‐lesional
PASI: Psoriasis Area and Severity Index
qPCR: quantitative polymerase chain reaction
RNA: ribonucleic acid
TNF: tumour necrosis factor
TRM: tissue‐resident memory T cell

## 1 Introduction

Psoriasis is a chronic inflammatory skin disorder characterised by erythematous, scaly plaques that can be both physically and emotionally distressing for individuals affected by the condition [1].

Many treatment options exist for psoriasis including topical treatments, systemic immunosuppressive treatments and biologics. Dead Sea climatotherapy (DSC) at Ein Gedi in Israel is a specialised intervention consisting of 4 weeks of sun, water and mud treatment. While this approach is used for a limited group of patients in Denmark, its application varies across the world [2]. It has been previously demonstrated that the treatment is highly effective in the short term and that among patients achieving complete skin clearance, defined as Psoriasis Area and Severity Index (PASI)‐100 response, the time in remission is approximately 3–6 months [3, 4, 5, 6].

While the exact cause of psoriasis is not fully understood, the central role of the interleukin (IL)‐23/IL‐17 axis in the pathogenesis of the disease is underscored by several highly efficient inhibitors of cytokines involved in these signalling pathways [7]. In addition, T cells, dendritic cells and neutrophils play a significant role in the development and progression of the disease, and neutrophilic microabscesses (Munro's microabscesses) are a pathognomonic sign of psoriasis [8]. Because DSC is a short effective treatment that allows for complete clearance among many patients, it can be used as a treatment modality to study the evolution of the various stages of psoriasis. The current study aimed to explore the cell profile in plaque psoriasis skin at relapse after complete skin clearance and how this profile differed from non‐lesional (NL) and chronic plaque lesional (LS) skin at baseline, in addition to completely cleared skin after treatment.

## 2 Methods

The patient cohort has previously been described elsewhere [3, 9, 10]. In short, we selected eight patients with plaque psoriasis who achieved PASI‐100 response from DSC at Ein Gedi in Israel. Patients were seen before DSC (baseline), at end of treatment (EOT) and at the first visible sign of psoriasis (relapse). Patient demographics can be seen in Table S1. The study was conducted in compliance with the Declaration of Helsinki, and signed informed consent was obtained from each patient prior to inclusion in the study (permission number: m‐20090102).

### 2.1 Biopsy Acquisition

Up to four punch biopsies were obtained from each patient at each visit. At baseline, a chronic psoriasis plaque that had been present for more than 3 months was defined as the target lesion, and a punch biopsy was acquired. At the EOT visit, another punch biopsy was taken from the same target lesion. At relapse, a punch biopsy was obtained from a visible element (see Table S1 for biopsy location). At baseline, a NL skin sample was acquired at least 5 cm from the target lesion.

### 2.2 Quantitative Immunohistochemistry

Prior to immunohistochemistry (IHC), the biopsies were paraffin‐embedded and sectioned into 4‐μm‐thick sections with a microtome, then deparaffinised and rehydrated in graded ethanol's. Antigen unmasking was performed by heating the sections in tris‐ethylene glycol tetraacetic acid buffer (pH 9) at preboiling temperature in a microwave oven. The antibodies used for immunohistochemical staining were CD11b, CD15, CD66b, CD207, myeloperoxidase (MPO) and neutrophil elastase (NE). See Table S2 for information on vendor, dilution, incubation time and clones used. Some sections were incubated without a primary antibody as negative controls. For detection, the Quanto Detection System was used (cat. No. TL‐060‐QHD, ThermoFisher Scientific, Waltham, MA, USA). Stained slides were digitised with a whole slide digital pathology scanner (NanoZoomer 2.0‐HT, RRID:SCR_021658, Hamamatsu Photonics K.K), using a 20× objective. Quantitative analysis was performed using the QuPath open‐source software (QuPath, RRID:SCR_018257, v0.5.1). The analysis was performed as previously explained with a dermal area constrained to the first 500 μm below stratum basale [11]. Stained cells were quantified separately in the epidermal and dermal compartments using the positive cell detection tool and were manually revised and corrected. All cell counts are reported as normalised to epidermal length.

### 2.3 RNA Purification

The snap‐frozen biopsies were submerged in RNAlater‐ICE (cat. No. AM7030, ThermoFisher Scientific) to thaw overnight at −20°C. Once thawed, the biopsies were added to SV RNA lysis buffer with β‐mercaptoethanol (SV Total RNA Isolation System, cat. No. Z3101; Promega, Madison, WI, USA) and homogenised using TissueLyser (RRID:SCR_025420, Qiagen, Hilden, Germany). The subsequent steps of RNA isolation were conducted per the manufacturer's instructions. Nanodrop 2000 (RRID:SCR_020309, ThermoFisher Scientific) was used to measure the RNA quantity and quality.

### 2.4 Bulk RNA Sequencing

Paired‐end RNA sequencing was performed by Eurofins Genomics Europe Sequencing GmbH (Konstanz, Germany). The libraries were prepared with NEBNext Ultra II Directional RNA Library Prep Kit and sequenced on the Illumina NovaSeq 6000 Sequencing System (RRID:SCR_016387) to generate at least 20 million read pairs. FastQC (RRID:SCR_014583, v0.12.0) was used for the quality control of the raw sequence data [12]. Reads were aligned to the human reference genome (GRCh38.p14 primary assembly) with the R package Subread (RRID:SCR_009803, v2.10.3) [13]. Uniquely aligned unambiguous reads were used for the subsequent analyses. Lowly expressed genes with fewer than five samples with normalised counts below five were filtered out. The data were variance stabilising transformed for downstream analyses, such as principal component analysis and heatmaps generated with pheatmap (RRID:SCR_016418, v.1.0.12) [14]. Gene‐level differential expression analyses were performed with DESeq2 (RRID:SCR_015687) (Wald test) [15]. Differentially expressed genes (DEGs) were identified using significance thresholds of false discovery rate (FDR)‐adjusted *p* values ≤ 0.05 and |log2(fold change)| ≥ 1. Gene Ontology (GO) enrichment analyses were performed with DAVID (RRID:SCR_001881) [16] and validated with ShinyGO (RRID:SCR_019213, v0.80) [17]. Gene set variation analyses were performed with the R package GSVA (RRID:SCR_021058, v1.46.0) using variance stabilising transformed normalised dataset [18]. Reactome gene sets were downloaded from The Molecular Signatures Database (RRID:SCR_016863, using msigbr v7.5.1) [19]. Gene set enrichment analysis (GSEA) was performed with fsgea (RRID:SCR_020938, v.1.28.0) using the genes from the differential expression analysis ranked by log2(fold change) *−log10(*p* value) [20]. Cell type enrichment analyses were performed with the xCell webtool using TPM (transcripts per million kilobase) normalised datasets, which generates enrichment scores for 64 cell types based on gene expression data [21].

### 2.5 Reverse Transcription Quantitative Real‐Time PCR

cDNA was generated with TaqMan Reverse Transcription Reagents (ThermoFisher) and Peltier Thermal Cycler‐200 (RRID: SCR_025421, MJ Research Inc., Waltham, MA, USA) following the manufacturer's instructions. Real‐time PCR was performed with 15 ng of cDNA (20 μL reactions in triplicates), on the StepOnePlus Real‐Time PCR System (RRID:SCR_015805, ThemoFisher) with TaqMan Universal PCR Master Mix Cat (cat. No. 4304437, ThemoFisher) and primers/probes (see Table S3). The real‐time PCR included two initial steps (2 min at 50°C and 10 min at 95°C) followed by 40 cycles (15 s at 95°C and 1 min at 60°C). Normalised target gene expression values were calculated with the relative standard curve method using StepOne Software (RRID:SCR_014281, v2.1) and RPLP0 as the reference gene [22].

## 3 Results

### 3.1 Relapsed Skin Treated With Dead Sea Climatotherapy Shows Heightened Activation of Key Immune Pathways Similar to Baseline Lesional Skin

**FIGURE 1:**
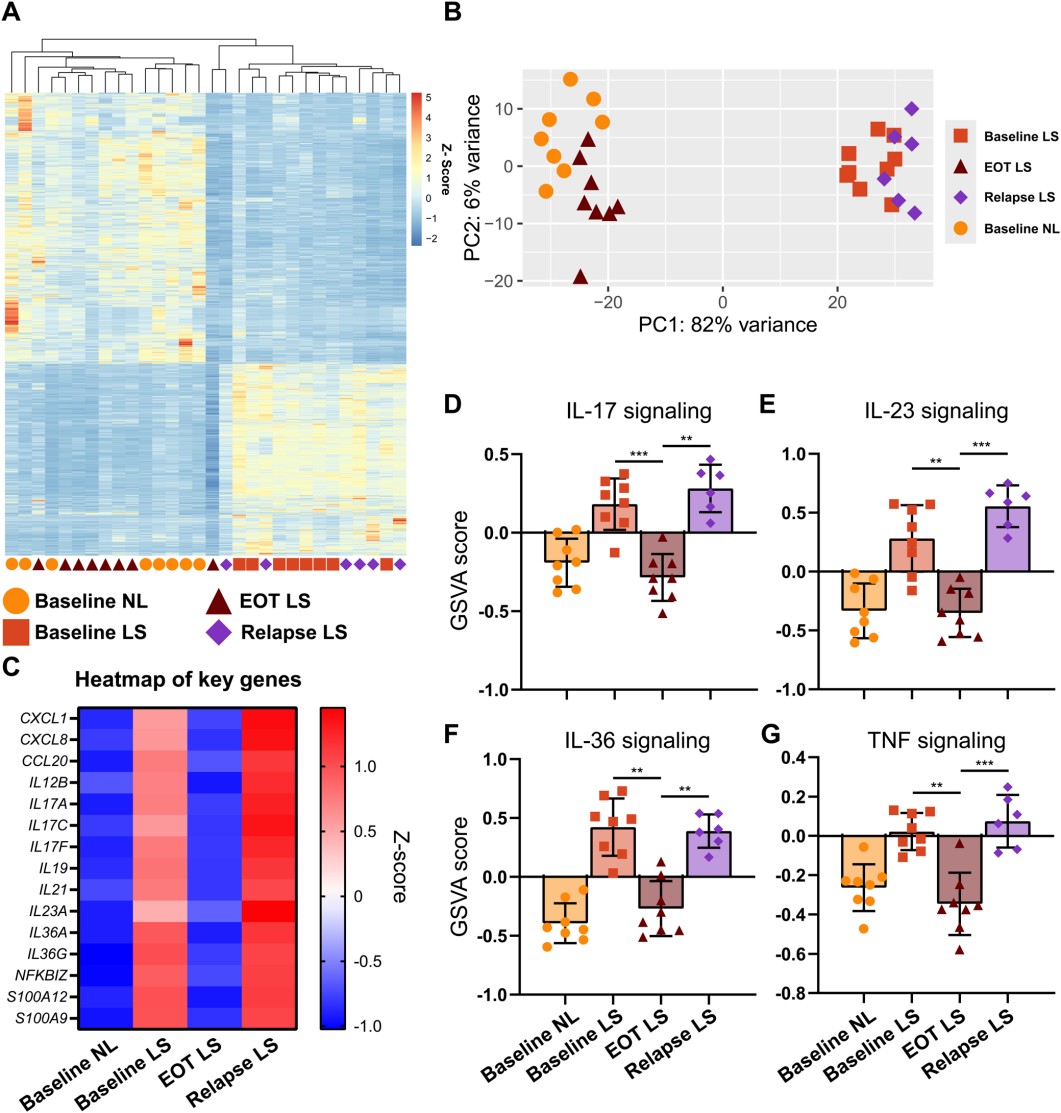
Transcriptomic analysis of the skin samples. (A) Semi‐supervised clustering and heatmap of differentially expressed genes in relapsed psoriasis skin compared with non‐lesional skin. The colours in the heatmap signify high (red) or low (blue) expression of a particular gene across samples (*z*‐score values). (B) Principal component analysis (PCA) plot of all the samples. (C) Heatmap showing the normalised expression of selected key psoriasis signature genes from the different visits. D‐G. Gene set variation analysis (GSVA) of key Reactome genesets at the different visits including (D) interleukin (IL)‐17 signalling, (E) IL‐23 signalling, (F) IL‐36 signalling and (G) tumour necrosis factor (TNF) signalling. Mean ± SD. Mixed effects analysis with post hoc Šidák. *p < 0.05, **p < 0.01, ***p < 0.001, ****p < 0.0001.

**FIGURE 2:**
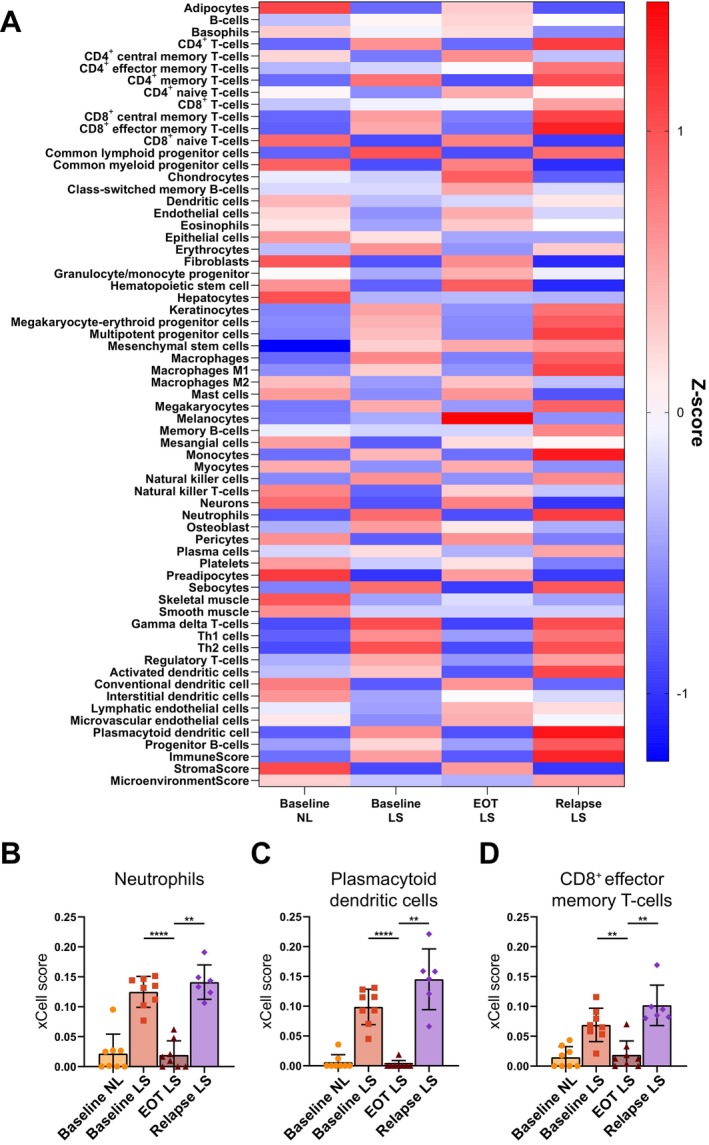
Cell type enrichment analysis from bulk transcriptome from the different visits. (A) Cell type enrichment analysis (CTEA) from bulk transcriptome from the different visits. The colours in the heatmap signify high (red) or low (blue) expression of a particular cell type across timepoints (*z*‐scaled values). (B) CTEA showing the neutrophil cells. (C) CTEA showing the plasmacytoid dendritic cells. (D) CTEA showing CD8^+^ effector memory T cells. Mean ± SD. Mixed effects analysis with post hoc Šidák. *p < 0.05, **p < 0.01, ***p < 0.001, ****p < 0.0001.

To characterise the molecular profile of the skin, we first performed bulk RNA transcriptome analysis of all the skin samples. The semi‐supervised clustering and heatmap showed that the samples separated according to predicted disease activity, that is, relapse LS grouped with baseline LS and baseline NL grouped with EOT LS (Figure 1A). This was corroborated by the PCA plot showing a clear separation of the groups (Figure 1B). By selecting only a few key psoriasis signature genes, there was a non‐significant trend towards an increased disease activity in skin at relapse compared with that of baseline LS (Figure 1C), including IL‐17, IL‐23, IL‐36 and TNF signalling, which are known to be heavily involved in the pathogenesis of psoriasis. Similarly, GSVA pathway analysis showed a tendency towards increased activity of IL‐17, IL‐23 and TNF signalling in relapse LS skin compared with baseline LS (Figure 1D–G). We next looked at the cell signatures from the different visits (Figure 2A). Several cell types were significantly downregulated following DSC and subsequently highly upregulated upon relapse, including neutrophils, plasmacytoid dendritic cells and T cells (Figure 2B–D). This suggested that DSC is highly effective at clearing the skin of inflammatory activity and cells, but also that these cells rapidly return in the de novo plaque at relapse.

### 3.2 Neutrophils Are Among the Earliest Immune Cell Populations to Be Significantly Upregulated During Relapse Following Dead Sea Climatotherapy

**FIGURE 3:**
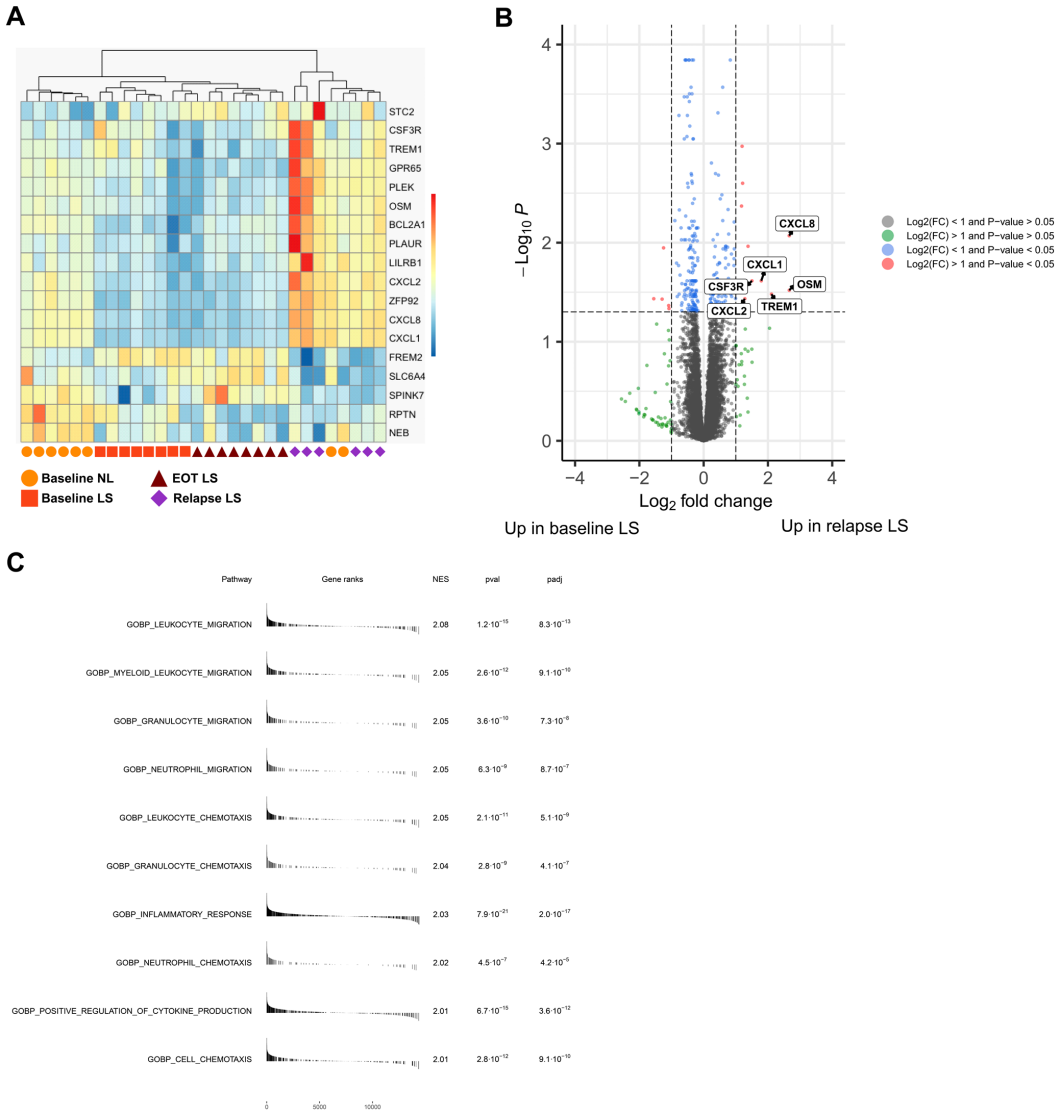
Transcriptomic analysis of baseline lesional and relapse lesional skin. (A) Semi‐supervised clustering and heatmap of all differentially expressed genes (DEGs) between relapse and baseline lesional skin. The colours in the heatmap signify high (red) or low (blue) expression of a particular gene across samples (*z*‐score values). (B) Volcano plot comparing relapsed and baseline lesional skin. Red dots denote differentially expressed genes (FDR‐adjusted *p* value < 0.05 and [|log2(fold change)| > 1]). Neutrophil‐related DEGs are highlighted. (C) Gene set enrichment analysis (GSEA) showing the top 10 upregulated gene sets in relapse LS versus baseline LS skin.

Having investigated the overall signature of all the skin samples, we next focused on the baseline LS versus relapse LS samples as these samples signify the chronic versus the de novo plaque. The heatmap of DEGs comparing relapse LS with baseline LS showed that the samples mostly separated according to predicted disease activity, that is, baseline LS separated from relapse LS (Figure 3A). Many of the upregulated DEGs, including *OSM*, *CXCL8*, *TREM1*, *CXCL1*, *CSF3R*, *BCL2A1* and *CXCL2*, were related to the activation of neutrophils (Figure 3B). The GSEA analysis showed that the most upregulated Gene Ontology biological process terms were related to leukocyte and neutrophil migration and chemotaxis (Figure 3C). Taken together, these results suggested that neutrophil activation is implicated in the early pathogenic events in the de novo plaque after complete skin clearance from DSC.

### 3.3 Real‐Time Quantitative Polymerase Chain Reaction Analysis Confirmed the RNA‐Seq Findings

To confirm the results obtained by RNA‐seq, we performed qPCR to detect and quantify *CXCL1*, *CXCL2*, *CXCL8*, *CSFR3*, *OSM* and *TREM1*, in addition to the psoriasis‐associated genes *IL‐17A* and *IL‐23*. A high concordance between the RNA‐seq and the qPCR data was observed, with increased expression in relapse LS compared with baseline LS; however, these trends did not reach statistical significance (Figure S1).

### 3.4 Immunohistochemistry Showed Abundant Quantities of Neutrophils at Baseline and in Relapsed Psoriasis Skin but Almost None in Non‐Lesional and Visually Clear Skin

**FIGURE 4:**
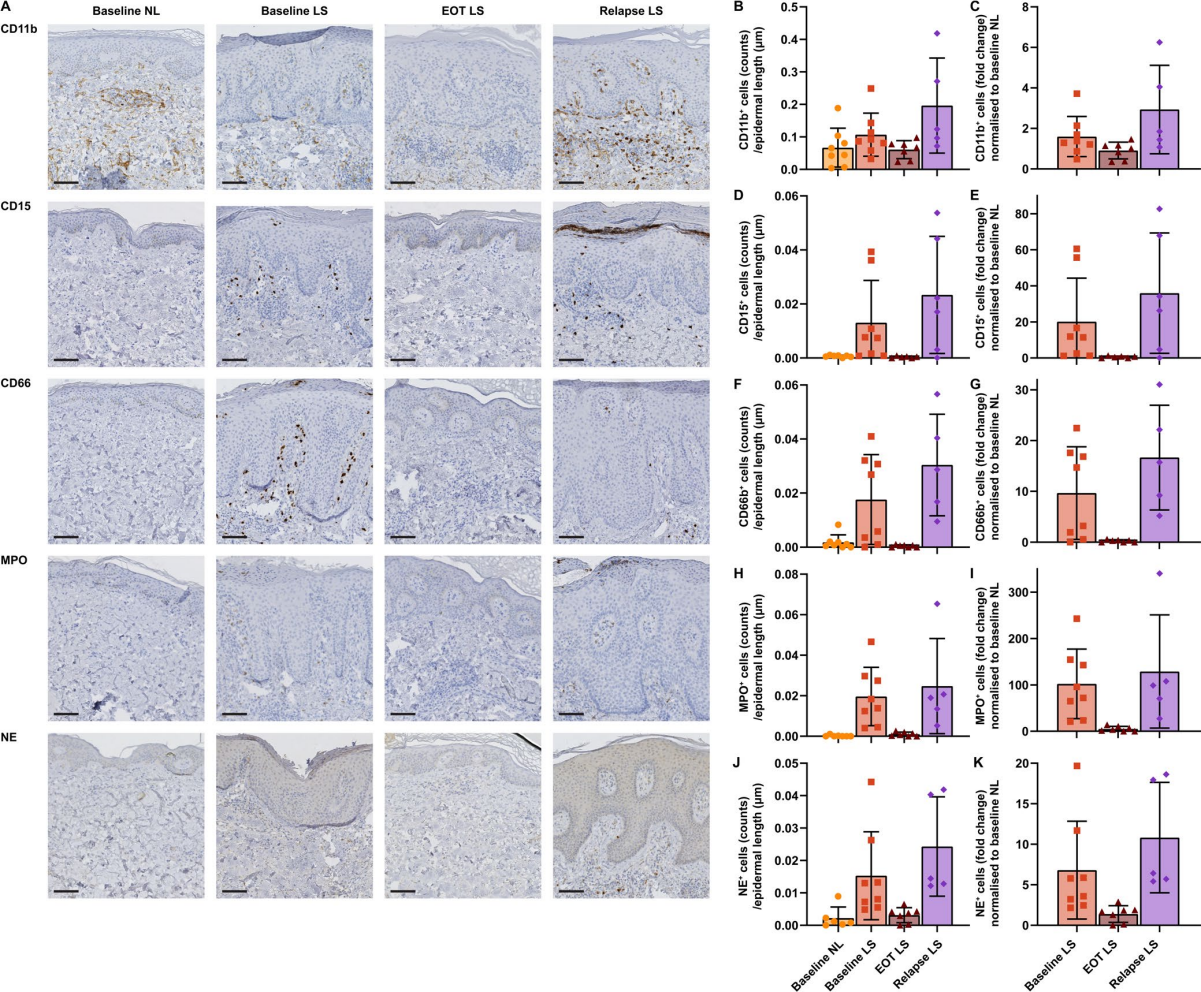
Results from quantitative immunohistochemistry of a group of selected neutrophil markers. (A) Immunohistochemistry of CD11b, CD15, CD66b, myeloperoxidase (MPO) and neutrophil elastase (NE). (B) CD11b^+^ cell counts from all the visits. (C) CD11b^+^ cell counts normalised to baseline NL depicted as fold change. (D) CD15^+^ cell counts from all the visits. (E) CD15^+^ cell counts normalised to baseline NL depicted as fold change. (F) CD66^+^ cell counts from all the visits. (G) CD66^+^ cell counts normalised to baseline NL depicted as fold change. (H) MPO^+^ cell counts from all the visits. (I) MPO^+^ cell counts normalised to baseline NL depicted as fold change. (J) NE^+^ cell counts from all the visits. (K) NE^+^ cell counts normalised to baseline NL depicted as fold change. Mixed effect analysis showed no significant differences between visits. Mean ± SD. Size bar = 100 μm.

To further investigate the extent of neutrophil infiltration in the skin, we next performed quantitative IHC. The same trends were observed for all analysed neutrophil markers (CD11b, CD15, CD66b, MPO and NE) (Figure 4). Baseline LS skin showed higher neutrophil cell infiltration compared with baseline NL skin. Between the three LS groups, EOT LS skin showed the lowest cell counts which were comparable with baseline NL levels. Interestingly, there was a trend towards higher neutrophil cell counts in relapse LS skin, surpassing the mean cell counts observed in baseline LS (Figure 4A,C,E,G). However, no significant differences between the visits were observed.

The fold change of cell counts was calculated from baseline NL for all LS samples, revealing the same trend (Figure 4B,D,F,H). These changes were most pronounced when analysing MPO^+^ cells and most discrete in CD11b^+^.

Langerhans cells, delineated mostly by the marker CD207 (Langerin), are known to be involved in antigen presentation and signal transduction [23]. In addition, *CD207* is one of the most rapidly responding genes after the initiation of adalimumab treatment [24]. To analyse the interaction between Langerhans cells and neutrophils, a double staining for markers CD207 and MPO was performed (Figure S2). CD207^+^ cell counts including fold changes compared with baseline NL appeared unchanged throughout the visits (Figure S2A,B). To further characterise the immune activity of these Langerhans cells, we quantified the fraction of these CD207^+^ cells that appeared to be in direct contact with MPO^+^ cells (Figure S2C). No contacts were observed in any of the baseline NL or EOT LS skin samples, but interaction was observed in half of the baseline LS and relapse LS skin samples. Although not statistically significant, the fraction of CD207^+^ cells in direct contact with MPO^+^ cells appeared highest at relapse LS. Conversely, the fraction of MPO^+^ cells in contact with CD207^+^ cells was similar at both baseline LS and relapse LS (Figure S2D). This suggested that a potential interaction between Langerhans cells and neutrophils existed in active psoriasis lesions.

## 4 Discussion

This study aimed to investigate the gene expression of the initial upregulated genes in relapsed psoriasis skin after complete clearance with DSC treatment. DSC enables rapid and complete skin clearance in many patients, yet psoriasis often reappears upon cessation of treatment. Psoriasis progression is believed to involve an initiation phase, followed by a chronic phase, and lastly a relapse‐remitting phase, potentially driven by localised disease memory in cells, such as tissue‐resident memory T cells (TRMs) or by epigenetic changes in skin cells [25, 26, 27, 28]. Consequently, DSC as a treatment model allows for the study of many phases of psoriasis.

In this study, we observed neutrophils to be a key cell type in the de novo plaque and may thus be one of the first cells to drive the progression of psoriasis. Traditional neutrophil markers such as NE, MPO, CD11b, CD15 and CD66b may thus be feasible targets for alleviating psoriatic symptoms. A previous study has been conducted on early‐phase (less than 1 month from debut) psoriasis patients with papular lesions [29]. Here, it was observed that the cytokines IL‐1β and IL‐8 were significantly increased in new‐onset psoriasis. Furthermore, immunohistochemistry showed abundant numbers of CD11b^+^, CD11c^+^, CD14^+^, CD163^+^ and plasmacytoid dendritic cells in early‐phase new‐onset psoriasis. IL‐1β plays an important role in autoinflammation [30], and by using confocal microscopy, IL‐1β was identified to be produced primarily by CD66b^+^ neutrophils [29]. It has been hypothesised that the early phase of psoriasis is characterised by the presence of neutrophils [31]. The de novo plaque in this study thus resembles changes seen in new‐onset psoriasis. Similar to plaque psoriasis, generalised pustular psoriasis (GPP) is characterised by a marked infiltration of neutrophils and the involvement of IL‐36‐related cytokines. Although both IL‐1β and IL‐36 are implicated in the pathogenesis of plaque psoriasis, their quantities are higher in GPP, where the IL‐36 signalling pathway plays a pivotal role [32]. We also observed that the IL‐36 pathway was highly upregulated at relapse, suggesting a role of this pathway in the relapse of psoriasis.

Several upregulated DEGs observed in relapse LS compared with baseline LS were related to inflammation, in particular neutrophil activation. OSM can be produced by neutrophils, and OSM has been shown to induce IL‐4 and suppress the T‐helper 1‐type and IL‐1‐responsive signals [33, 34]. The IL‐17 pathway‐associated cytokines CXCL1, CXCL8 are produced by keratinocytes and attract neutrophils [35]. TREM1 is found co‐expressed on neutrophils and has been found to be involved in the early phase of psoriasis [36]. CSF3R is involved in the differentiation and proliferation of myeloid progenitor cells into neutrophils [37]. BCL2A1 is implicated in chronic inflammatory disorders by recruiting and stabilising myeloid cells [38, 39]. CXCL2 is produced by neutrophils and helps in guiding neutrophils to extravasation sites in inflamed tissues [40]. Thus, many of the DEGs found are encoding proteins involved in the recruitment of neutrophils to the site of inflammation following inflammatory signals (such as IL‐17 and TNF) and the subsequent production and release of large amounts of reactive oxygen species, possibly contributing to psoriasis [41]. This may explain the rapid epidermal clearance of neutrophils observed in IL‐17A inhibition [42].

This study is subject to several limitations, including the small sample size. In addition, because there was a small lag in time from first experiencing symptoms to biopsy acquisition, there is a risk that the very early phase of psoriasis may be driven by other immune‐mediated factors than those found in this study. Nonetheless, our findings using DSC as a treatment model suggest that neutrophils are among the earliest immune cells activated during psoriatic relapse after complete skin clearance. This observation may be important for our understanding of psoriasis pathogenesis and may identify new treatment options in psoriasis. Targeting psoriasis in the early stages may inhibit the chronification of the disease, thus potentially providing improved disease control.

## Author Contributions

Conceptualisation: T.E., H.B.A., L.I., C.J.; data curation: T.E., H.B.A., E.B., I.M.R.; formal analysis: T.E., H.B.A., E.B., I.M.R.; funding acquisition: T.E.; investigation: T.E., H.B.A., E.B., I.M.R., A.B.; methodology: T.E., H.B.A., E.B., C.J.; project administration: T.E., H.B.A., C.J.; resources: T.E., H.B.A., T.S., A.B., L.I., C.J.; software: H.B.A.; supervision: T.S., A.B., L.I., C.J.; validation: T.E., H.B.A.; Visualisation: T.E., H.B.A., E.B., I.M.R.; writing – original draft: T.E., H.B.A., E.B., I.M.R., T.S., L.I., C.J.; writing – review and editing: T.E., H.B.A., E.B., I.M.R., T.S., A.B., L.I., C.J.

## Conflicts of Interest

T.E. reports conducting investigator‐initiated trials for LEO Pharma and Johnson & Johnson in addition to having received presentation honoraria from LEO Pharma and Bristol‐Myers Squibb, all without relevance to the current manuscript.

## Supporting information


Data S1

## Data Availability

The datasets used and/or analysed during the current study are available at the Gene Expression Omnibus repository (https://www.ncbi.nlm.nih.gov/geo, GSE: GSE285522) and the corresponding author on reasonable request. Any datasets generated will be made available prior to publication.
