# Supplementary material for: Early Neutrophil Activation in Psoriatic Skin at Relapse Following Dead Sea Climatotherapy
Source: Exp Dermatol. 2025 Apr 3;34(4):e70094. doi: 10.1111/exd.70094 (PMC11969059; doi:10.1111/exd.70094)
Supplement: Supplementary file 1 — Data S1 [file EXD-34-e70094-s001.zip › exd70094-sup-0001-manuscript_supplementary_2024_11_30.docx]

**Supplementary:**

**Table S1. Patient demographics and biopsy site locations.**

| Patient no. | Age | Sex | PASI  Baseline | PASI  EOT | PASI  Relapse | Biopsy site at  baseline and EOT | Biopsy site at relapse |
| --- | --- | --- | --- | --- | --- | --- | --- |
| 1 | 57 | F | 11.5 | 0 | 9.3 | Left gluteal | Right gluteal region |
| 3 | 44 | M | - | 0 | 4.5 | Left lower back | Left lower back region |
| 4 | 38 | M | 10.6 | 0 | 2.7 | Left hip | Left hip |
| 8 | 52 | M | 11.6 | 0 | - | Right lower back | Right lower back |
| 9 | 45 | M | 23.6 | 0 | 3 | Left upper arm | - |
| 13 | 57 | F | 17 | 0 | - | Right gluteal | Right gluteal |
| 14 | 50 | M | 18.7 | 0 | - | Left upper arm | - |
| 19 | 62 | M | 11.4 | 0 | 5.6 | Left gluteal | - |

†Abbreviations: PASI, Psoriasis Area and Severity Index; EOT, End of treatment.

| Marker | Development stage | Clone | Lot | Dilution | Incubation length | Vendor | Isotype | Cat Number | RRID |
| --- | --- | --- | --- | --- | --- | --- | --- | --- | --- |
| CD11b | Activation, maturation | EP45 | 230101862 | 1:1000 | Overnight | Abcam | Rabbit Monoclonal,  IgG_κ_ | AC-0043RUO | Abcam Cat# AC-0043RUO, RRID:AB_10999891 |
| CD15 | Activation, maturation | MMA | 45963 | 1:100 | Overnight | Cell Marque | Mouse Monoclonal,  IgM | 115M-14 | Cell Marque Cat# 115M-14, RRID:AB_1157919 |
| CD66b | Activation | BLR111H | YK4138991 | 1:75 | Overnight | Thermo Fisher Scientific | Rabbit Monoclonal, IgG | MA544413 | Thermo Fisher Scientific Cat# MA5-44413, RRID:AB_2926543 |
| CD207 | Langerhans cell | 12D6 | 70437 | 1:100 | 30 min | Cell Marque | Mouse Monoclonal, IgG_2b/κ_ | 392M-15 | Millipore Cat# 392M-1, RRID:AB_2889342 |
| MPO | Immature and mature stages | SP72 | 46344 | 1:200 | Overnight | Cell Marque | Rabbit Monoclonal, IgG_1_ | 289R-14 | Cell Marque Cat# 289R, RRID:AB_2892655 |
| Neutrophil Elastase | Migration | EPR7479 | GR3335199-1 | 1:600 | Overnight | Abcam | Rabbit Polyclonal, IgG | AB-68672 | Abcam Cat# ab68672, RRID:AB_1658868 |

**Table S2. List of antibodies used for immunohistochemistry.**

†Abbreviations: CD, cluster of differentiation; MPO, myeloperoxidase; NE, neutrophil elastase; RRID, research resource identifier.

**Table S3. List of gene expression assays used for the experiment.**

| Gene name | Ensemble ID | TaqMan^®^ Gene Expression Assay |
| --- | --- | --- |
| *CSF3R* | ENSG00000119535 | Hs01114420_m1 |
| *CXCL1* | ENSG00000163739 | Hs00236937_m1 |
| *CXCL2* | ENSG00000081041 | Hs00601975_m1 |
| *CXCL8* | ENSG00000169429 | Hs00174103_m1 |
| *IL-17A* | ENST00000648244 | Hs00174383_m1 |
| *IL-23* | ENSG00000110944 | Hs00372324_m1 |
| *OSM* | ENSG00000099985 | Hs00171165_m1 |
| *TREM1* | ENSG00000124731 | Hs00218624_m1 |
| *RPLP0* | ENSG00000089157 | Hs99999902_m1 |

**Table S4. Overrepresentation GO pathway analysis of relapsed LS versus baseline LS.**

**Table S5. Overrepresentation KEGG pathway analysis of relapsed LS versus baseline LS.**

**Table S6. Gene set enrichment analysis (GSEA) of relapsed LS versus baseline LS using GO BP terms.**

**
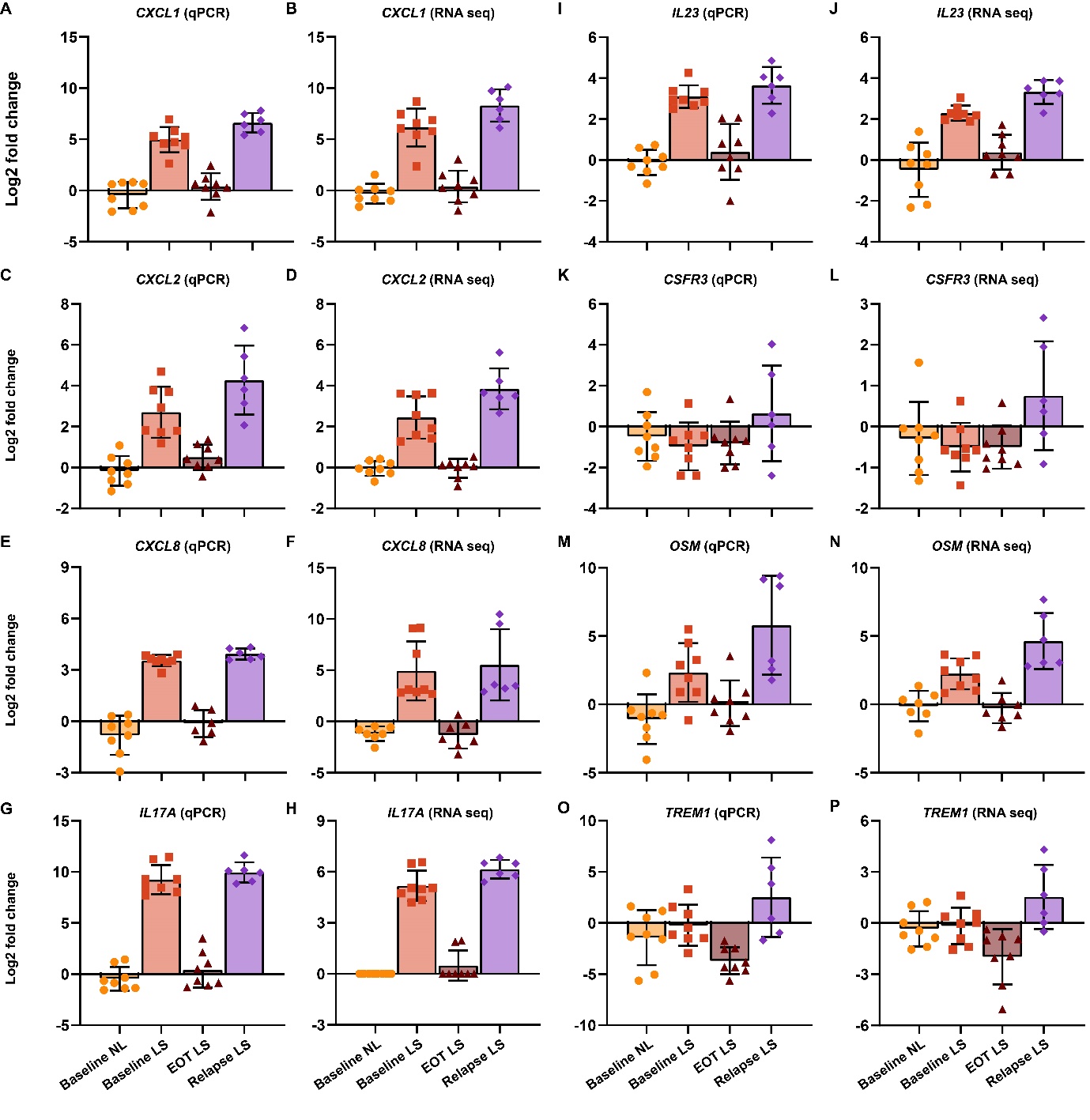
**

**Figure S1. Results from quantitative qPCR and RNA sequencing.**

A. *CXCL1* counts from qPCR. B. *CXCL1* counts from RNA-seq. C. *CXCL2* counts from qPCR. D. *CXCL2* counts from RNA-seq. E. *CXCL8* counts from qPCR. F. *CXCL8* counts from RNA-seq. G. *IL-17A* counts from qPCR. D. *IL-17A* counts from RNA-seq. I. *IL-17A* counts from qPCR. J. *IL-17A* counts from RNA-seq. K. *IL-17A* counts from qPCR. L. *IL-17A* counts from RNA-seq. M. *OSM* counts from RNA-seq. N. *OSM* counts from qPCR. L. *IL-17A* counts from RNA-seq. O. *TREM1* counts from qPCR. L. *TREM1* counts from RNA-seq. O. *TREM1* counts from qPCR. P. *TREM1* counts from RNA-seq. Log2 fold change of normalized counts depicted. Mixed effect analysis showed no significant differences between visits. Mean ± SD. Mixed effect analysis showed no significant differences between visits.


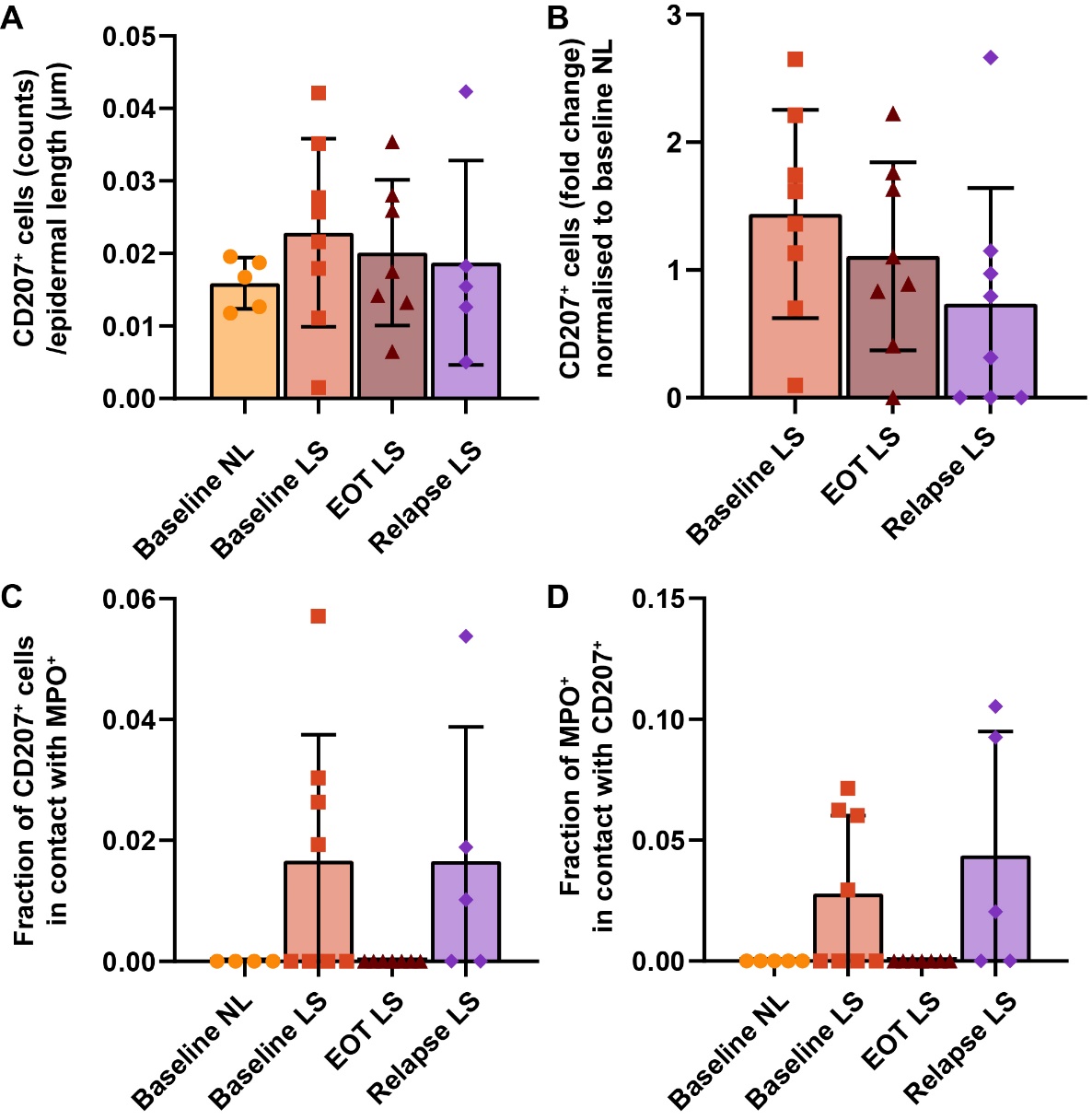
**Supplementary Figure 2.** **CD207^+^ cell counts and colocalizations from all the visits.**

A. CD207^+^ cell counts from all the visits. B. CD207^+^ cell counts normalized to baseline NL depicted as fold change. C. Fraction of CD207^+^ cells in contact with MPO^+^ cells. D. Fraction of MPO^+^ cells in contact with CD207^+^ cells. Mixed effect analysis showed no significant differences between visits.
